# Classification of severe obstructive sleep apnea with cognitive impairment using degree centrality: A machine learning analysis

**DOI:** 10.3389/fneur.2022.1005650

**Published:** 2022-08-25

**Authors:** Xiang Liu, Yongqiang Shu, Pengfei Yu, Haijun Li, Wenfeng Duan, Zhipeng Wei, Kunyao Li, Wei Xie, Yaping Zeng, Dechang Peng

**Affiliations:** ^1^Department of Radiology, the First Affiliated Hospital of Nanchang University, Jiangxi, China; ^2^Big Data Center, the Second Affiliated Hospital of Nanchang University, Jiangxi, China; ^3^Department of PET Center, the First Affiliated Hospital of Nanchang University, Jiangxi, China

**Keywords:** obstructive sleep apnea, resting-state functional magnetic resonance imaging, degree centrality, machine learning, mild cognitive impairment

## Abstract

In this study, we aimed to use voxel-level degree centrality (DC) features in combination with machine learning methods to distinguish obstructive sleep apnea (OSA) patients with and without mild cognitive impairment (MCI). Ninety-nine OSA patients were recruited for rs-MRI scanning, including 51 MCI patients and 48 participants with no mild cognitive impairment. Based on the Automated Anatomical Labeling (AAL) brain atlas, the DC features of all participants were calculated and extracted. Ten DC features were screened out by deleting variables with high pin-correlation and minimum absolute contraction and performing selective operator lasso regression. Finally, three machine learning methods were used to establish classification models. The support vector machine method had the best classification efficiency (AUC = 0.78), followed by random forest (AUC = 0.71) and logistic regression (AUC = 0.77). These findings demonstrate an effective machine learning approach for differentiating OSA patients with and without MCI and provide potential neuroimaging evidence for cognitive impairment caused by OSA.

## Introduction

Obstructive sleep apnea (OSA) is a common sleep disorder characterized by repeated airflow stoppages caused by partial obstruction of the upper airway during sleep, affecting approximately 14% of adult men and 5% of adult women (1, 2). Recurrent upper airway obstruction in OSA patients results in intermittent hypoxia, fragmented sleep, and excessive day-time sleepiness (3). Furthermore, OSA has been shown to be associated with mild cognitive impairment (MCI), especially in older adults (4). Currently, the research on OSA and MCI is still in its infancy; however, there is some evidence suggesting that oxidative stress and endothelial function damage caused by intermittent hypoxia are related to cognitive impairment (5). However, the neuroimaging mechanisms involved in the association between OSA and MCI are not fully understood, and the assessment of OSA cognitive impairment is challenging to some extent.

With the continuous development of imaging technology, researchers have been involved in the study of MCI, brain function, and brain structure progressively. Reportedly, OSA patients have multiple brain abnormalities related to cognitive dysfunction apparent in regions such the cerebellum, insula, temporal area, and hippocampus (6–9). Resting state functional magnetic resonance (fMRI) reflects brain function under *in vivo* physiological and pathological conditions through resting oxygen-dependent changes. In the resting state, neurons in the brain exhibit spontaneous activity that is transmitted to other neurons, forming a complex network of functions. DC can explore the characteristics of the whole brain functional connection at the voxel level (10), complete the construction of the whole brain functional network, explore the functional community within the functional connection group (11), and avoid the influence of subjective seed point selection. Simultaneously, DC does not require prior prediction, making it more suitable for exploring neural correlations of dimensional and classified phenotypic data (12). Our previous study showed that the DC changes in the bilateral posterior cerebellar, frontal, temporal, and insula lobes before and after CPAP therapy confirmed the high overlap between the reversed brain region and the initial injury brain region, objectively reflecting the effectiveness of CPAP therapy (13). In another Alzheimer's study, patients who recovered from MCI had lower DC in the right lower cerebellum and higher DC in the left superior medial frontal gyrus and left inferior temporal gyrus compared with healthy participants, suggesting that loss of function in local brain structures could be compensated for by enhanced function in surrounding areas (14). Enabled by the high sensitivity and repeatability of DC technology (15), the application of DC to cognitive disability-related diseases to explore the reversible potential physiological mechanism of neural network injury and brain injury has become more frequent (16, 17).

Machine learning is widely used in binary classification because of its parallelism, self-organization, adaptive learning ability, and robustness (18). Common classification methods in data mining and machine learning include artificial neural network, logistic regression (LR), random forest (RF), and support vector machine (SVM)(19–22). Khatri et al. performed diagnostic classification of MCI patients and healthy people based on multimodal MRI (ReHo, fALFF, ALFF, DC) and hippocampal and amygdala volumes, and compared the classification efficiency of various machine learning methods. Eventually, they achieved good classification performance, with SVM as the best classifier (AUC 94.03%, accuracy 92.45%) (23). Bigham et al. used diffusion tensor imaging for diagnostic classification of MCI patients and healthy people in combination with a fast correlation filter for feature screening of high-dimensional data and obtained a good SVM classification feature model with 83.3% accuracy and 80.7% sensitivity (24).

Based on those findings, in this study, we assume that a variety of machine learning methods can be used to construct classification models (including SVM, RF and LR) through DC features, and can effectively identify patients with cognitive impairment from OSA patients. The objectives of this study were as follows: 1. DC was used to detect OSA patients with MCI and OSA patients without MCI, while the LASSO regression method was used to screen out the most characteristic brain regions, 2. A variety of machine learning methods (SVM, LR and LR) were compared simultaneously to build the optimal performance model.

## Material and methods

All OSA patients were diagnosed with obstructive sleep apnea in the sleep monitoring room of the First Affiliated Hospital of Nanchang University's Department of Respiratory Medicine, between August 2017 and June 2022. The diagnosis of all patients was jointly determined by experienced respiratory physicians in accordance with the guidelines of the American Academy of Sleep Medicine (AASM) 2017 Clinical Practice Guidelines for adult obstructive sleep apnea (25). Inclusion criteria were as follows: apnea hypopnea index (AHI) > 15/h; All participants were right-handed, native Chinese speakers and aged 20 to 60. Exclusion criteria were as follows: (1) Sleep disorders other than OSA (e.g., insomnia, drowsiness); (2) Respiratory diseases, cardiovascular diseases, diabetes mellitus, hypothyroidism, central nervous system diseases, trauma, and other conditions that would explain an AHI > 15/h independent of OSA; (4) Alcohol or illicit drug abuse or current use of psychotropic substances; (4) Contraindications to MRI, such as claustrophobia; (5) Image artifact. A final 99 OSA patients were included in the analysis. We abide by the principles of the Declaration of Helsinki. This study was approved by the Medical Ethics Committee of the First Affiliated Hospital of Nanchang University [2020(94)]. Participants signed written informed consent documentation for this study.

### Research framework

**Figure 1:**
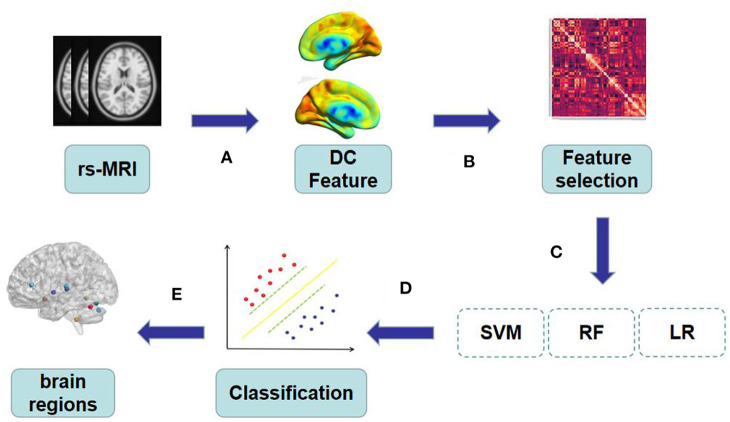
**(A)** The original rs-MRI was preprocessed and regions of interest were extracted by AAL template as features. **(B–C)** All the features extracted from DC were screened for feature correlation, and the minimum absolute contraction and Selection operator logic method was used for 10-fold cross verification to retain the features with non-zero coefficients. **(D)** The extracted features are trained by SVM, RF and LR to obtain the best model. **(E)** Visual mapping of brain regions according to the characteristics of the best model.

Our research framework is shown in Figure 1 and the specific steps are as follows:

### Polysomnography and neuropsychological assessment

Prior to polysomnography (PSG) monitoring, all participants were asked not to consume alcohol or coffee. All participants received an overnight PSG (from 10 p.m. to 6 a.m.), using the Respironics LE physiological monitoring system of (Alice 5 LE, Respironics, Orlando, FL, USA). PSG monitoring includes standard electrocardiogram, electro-ophthalmogram, electromyogram, electrocardiogram, body position, nasal and oral airflow, chest and abdominal respiratory movement, snoring, etc. Saturation of pulse oxygen (SpO_2_), sleep latency, total sleep time, sleep efficiency, sleep stage, awakening, and respiratory events were recorded (26). Obstructive apnea is described as a continuous 90% reduction in airflow, lasting > 10 seconds, with significant dyspnea. The apnea index (AHI) is the sum of apnea and hypopnea events occurring per hour during sleep.

All participants completed the MoCA cognitive scale assessment in a quiet state under the guidance of a professional neuropsychologist. Cognitive function was assessed using 11 MoCA items, which were examined in eight cognitive domains, including executive function, language, attention, computation, abstraction, naming, memory, and orientation. A MoCA score < 26 indicates cognitive impairment (27).

### MRI data acquistion

MRI images were collected for all participants in a 3.0 Tesla MRI scanner in our hospital's 8-channel phased array head coil (Siemens, Munich, Germany). Foam pads were used to reduce the patient's head movement, and earplugs were used to reduce scanner noise. Before the scan, all participants were required to close their eyes, stay awake and not engage in specific thinking activities. First, conventional MRI scan was performed, and conventional T1-weighted imaging was performed: Repetition time (TR) = 250 ms, echo time (TE) = 2.46 ms, Thickness = 5 mm, clearance = 1.5 mm, FOV = 220 x 220 mm, TR= 4,000 ms, TE = 113 ms, thickness = 5 mm, Clearance = 1.5 mm, FOV = 220 × 220 mm, slice = 19). Then, high-resolution T1-weighted MRI images of brain structures were obtained from each subject using brain volume sequences on the sagittal plane (TR = 1,900 ms, TE = 2.26 ms, thickness = 1.0 mm, gap = 0.5 mm, FOV = 250 × 250 mm, Matrix = 256 × 256, turn Angle = 9, slice = 176). Finally, in the axial plane (TR = 2,000 ms, TE = 30 ms, turn Angle = 90, thickness = 4.0 mm, clearance = 1.2 mm, rs-fMRI data), field of view = 230 × 230 mm 2, matrix size = 64 × 64, slice = 30), a total of 240 rs-fMRI images were recorded. Two experienced radiologists read the images to exclude lesions and motion artifacts visible to the naked eye.

### Data pre-process

Imaging data were examined with MRIcro software (www.MRIcro.com) to discard suboptimal data. Data were obtained from resting state using the Data Processing & Analysis for Brain Imaging toolkit (DPABI, Chinese Academy of Sciences, Beijing, China, http://rfmri.org/dpabi), based on statistical parameter mapping (SPM12, http://www.fil.ion.ucl.ac.uk/spm/software/spm12/) and MATLAB2018b (Math Works, Natick, MA, USA). First, the file format was converted from DICOM to NIFTI. Then, time layer correction and 3D head motion correction were carried out for the remaining time series. Participants with frame displacement > 2.5 standard deviations were excluded (28). Linear transformation was used to co-register structural images with functional images of each subject. Therefore, the new segmentation in SPM12 was used to segment structural images of all participants into white matter and cerebrospinal fluid. Then, the image space was normalized to the Montreal Neurological Institute (MNI) template and resampled to 3 × 3 × 3 mm^3^ voxels. Finally, linear regression was used with regression Friston 24 parameters, white matter signals, and cerebrospinal fluid signals from all voxel time series, after filtering using a time filter (0.01–0.08 Hz). Please refer to our previous study for more details (29).

### Voxel-level degree centrality

The default whole-brain gray matter template (61 × 73 × 61, 3 mm × 3 mm × 3 mm, 67,541 voxels) was extracted using DPABI software. Pearson correlation coefficients between arbitrary voxels of each subject are calculated in the default gray matter template. DC between voxels was calculated according to the following formula (10):


$$\begin{array}{l} Dc\left(i\right)=\overset{N}{\underset{j=1}{\sum }}rij\left(rij>r0\right) \end{array}$$

The correlation coefficient between voxel i and voxel j is expressed as r_ij_, and the correlation threshold used to eliminate weak correlation is called r0 (30). Then Fisher transform was used to transform the correlation coefficient into z-score graph to improve the normality. Finally, gauss was used to check the z-score graph with the maximum half-width and height of 6 mm for smoothing.

### Feature extraction and feature selection

After rs-fMRI data pretreatment, zDC of each brain region of the zDC map obtained by us were extracted based on automatic anatomical labeling (AAL) map (31), and 116 brain regions were selected as features.

Firstly, Pearson's correlation coefficient between each group of features was calculated, and 0.75 was set as the absolute correlation threshold. For feature pairs whose correlation was greater than the threshold value, the variables with higher average absolute correlation were deleted after comparing their average absolute correlation, weakening multicollinearity at a small cost. Then, we used the least absolute shrinkage and selection operator (LASSO) logic method for 10-fold cross validation (32), Alpha was searched in the range of 10^−6^ to 10^3^, with a step size of 10^0.2^, and the optimal Alpha value was selected as a cost function using the mean square error (MSE). Finally, non-zero coefficient features were selected to train the classification model. Feature selection is performed in Python 3.8.8 using the software package “scikit-learn” (33).

#### SVM

SVM is a supervised learning technique for partitioning and classification by searching for the optimal hyperplane. The algorithm was originally designed to solve the problem of binary classification. It has good generalization ability, can avoid dimensional disaster, and is widely used in neuroimaging and disease classification (34, 35).

#### RF

RF is a comprehensive learning algorithm that uses multiple decision trees for prediction. It is a vote on the predicted results of all decision trees (36). The variance of the model can be effectively reduced by constructing the training set with random sampling so that each feature is a part of the whole feature vector.

#### LR

The LR is a supervised machine learning classifier that predicts the likelihood of a target variable (37). This multivariable technique seeks to establish a functional relationship between many predictive variables and a single output. LR is a powerful maximum likelihood algorithm that can use discrete and continuous data sets to generate probabilities and classify new data.

### Classification

We construct three representative machine learning classification models, namely SVM, RF, and LR models, respectively. GridSearchCV is used for hyperparameter optimization, and a Leave-one-out cross-validation method and permutation test (5000 times) are used for model performance verification. We calculated the accuracy, sensitivity, and specificity of different models, and used the receiver operating characteristic (ROC) curve and area under the curve (AUC) to evaluate the performance of the models. The optimal model was selected, and Cohen's Kappa was used to evaluate the heterogeneity of test results. All selected DC features are weighted to quantify their contribution to the model.

### Statistics

For the demographic and clinical evaluation data, SPSS 23.0 software was first used for processing, and kolmogorov-Smirnov was used to test the normality of the data. Then, two-sample *t*-test was performed on the data conforming to normal distribution, and Mann-Whitney *U* test was performed on the non-normal distribution data. *P* < 0.05 was considered statistically significant. Chi-square test was adopted for the data of dichotomous variables, and *P* < 0.05 was considered statistically significant.

## Results

### Demographic and clinical characteristics

Summary of demographic and clinical characteristics of OSA patients in MCI group and nMCI group (Table 1). We found that there were significant differences in neck circumference and MoCA scale between MCI group and nMCI group, *P* < 0.05. There were no significant differences in age, education, BMI, waist circumference, AHI, Nadir SpO_2_, mean SpO_2_, Total sleep time, N1, N2, N3, SpO_2_ <90%, sleep efficiency and REM between the two groups (*P* > 0.05).

**Table 1:** General clinical scale.

	**MCI (*n* = 51)**	**nMCI (n = 48)**	***P* value**
Sex (M/F)	48/3	47/1	0.654
Age (year)^a^	38.47 ± 7.93	35.45 ± 8.76	0.076
Education (year)^b^	12.98 ± 2.33	13.78 ± 3.21	0.163
BMI (Kg/m^2^)^a^	27.26 ± 3.06	26.78 ± 4.20	0.514
Neck circumference (cm)^b^	41.17 ± 3.24	39.95 ± 2.77	0.048
Waistline (cm)^b^	99.19 ± 6.92	97.04 ± 15.18	0.361
AHI^a^	53.19 ± 23.12	49.58 ± 19.23	0.402
Nadir SpO_2_ (%)^b^	71.25 ± 12.52	68.31 ± 12.52	0.244
MSpO_2_ (%)^b^	92.38 ± 3.57	91.82 ± 5.13	0.530
Total sleep time (min)^b^	366.05 ± 112.36	379.20 ± 77.78	0.503
Sleep efficiency (%)^b^	80.01 ± 22.20	85.74 ± 12.14	0.118
N1(%)^b^	28.94 ± 17.26	25.16 ± 16.58	0.270
N2(%)^b^	39.32 ± 12.68	43.16 ± 15.09	0.174
N3(%)^b^	19.58 ± 14.68	21.39 ± 15.68	0.555
REM(%)^b^	15.45 ± 9.90	12.63 ± 8.71	0.137
SpO_2_ <90%^b^	24.34 ± 20.02	23.23 ± 16.36	0.764
MoCA^b^	22.23 ± 2.61	27.27 ± 1.16	<0.001

MCI, mild cognitive impairment; nMCI, no mild cognitive impairment; AHI, apnea hypopnea index; Nadir SpO_2_, minimum saturation of pulse oxygen; MSpO_2_, average saturation of pulse oxygen; REM, rapid eye movement; SpO_2_ <90%, percentage of total sleep time with oxygen saturation <90; ^a^, Student, t-test; ^b^, Mann-Whitney U-test.

### Feature selection

**Figure 2:**
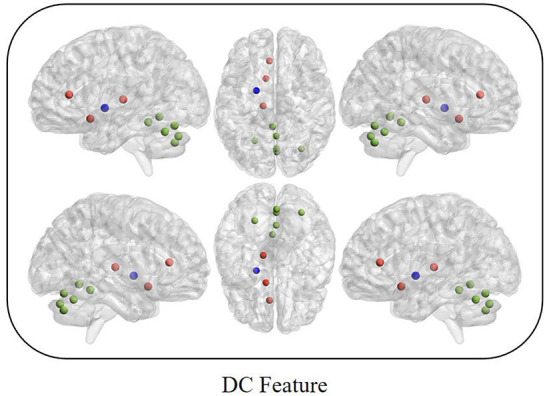
Red represents the default mode network; Blue represents the basal node network; Green represents the cerebellum network.

Between MCI and nMCI groups, we finally obtained 10 DC features through feature selection procedures, as shown in Figure 2, Table 2.

**Table 2:** The selected DC features set for discriminating the MCI from nMCI group.

**ID**	**Feature**	**Brain Network**	**MCI**		**nMCI**		**Weight**		
			**Mean**	**SD**	**Mean**	**SD**	**SVM**	**LR**	**RF**
1	Olfactory R	DMN	−0.095	0.292	−0.187	0.245	0.45695206	0.61365026	0.07962183
2	Cingulum Ant L	DMN	0.174	0.336	0.293	0.385	−0.45212804	−0.41221711	0.08364737
3	Pallidum L	Basal node network	−0.11	0.383	0.088	0.477	0.03777478	−0.02233599	0.07402411
4	Transverse temporal L	DMN	0.26	0.412	0.489	0.351	−0.28568111	−0.3511914	0.10909707
5	Cerebelum Crus1 R	Cerebellum network	0.059	0.280	−0.088	0.289	0.57115715	0.49502768	0.10136584
6	Cerebelum 4 5 L	Cerebellum network	−0.027	0.221	0.151	0.239	−0.3993657	−0.3675441	0.17269695
7	Vemis 1	Cerebellum network	−0.386	0.348	−0.258	0.272	−0.14545911	−0.44863276	0.09362268
8	Vemis 6	Cerebellum network	−0.123	0.280	0.001	0.335	−0.47500579	−0.24829838	0.07821442
9	Vemis 8	Cerebellum network	−0.419	0.310	−0.286	0.295	−0.37868619	−0.58716901	0.11912403
10	Vemis 10	Cerebellum network	−0.399	0.304	−0.503	0.284	0.56859686	0.84123316	0.08858571

DMN, default mode network; MCI, mild cognitive impairment; nMCI, no mild cognitive impairment; SVM, support vector machine; LR, logistic regression; RF, random forest.

### Classification efficiency

**Figure 3:**
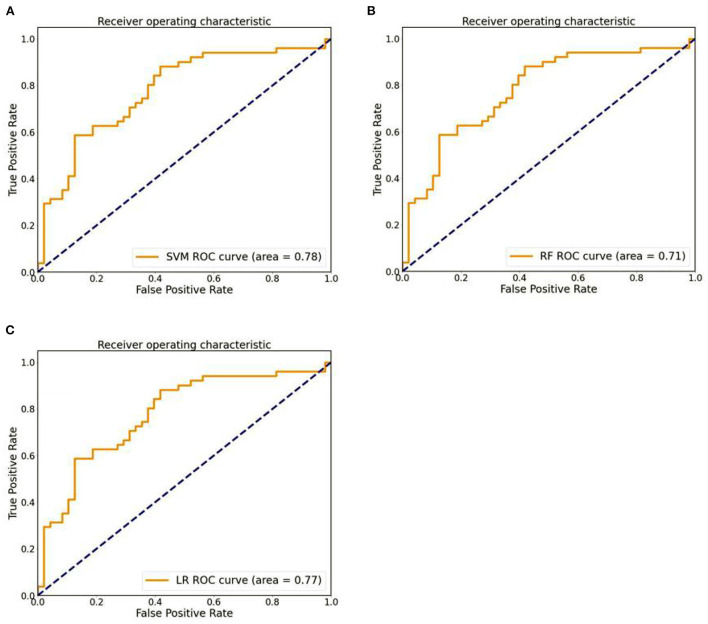
**(A)** The ROC curves of SVM models. **(B)** The ROC curves of RF models. **(C)** The ROC curves of LR models.

After the above feature screening method, 10 brain region DC features were selected, and the performance comparison of the three machine learning models was obtained after hyperparameter optimization and retention cross-validation, as shown in Table 3, Figure 3. The accuracy of SVM model was 0.71 and AUC was 0.78(sensitivity = 82.35%, specificity = 60.42%, *p*-value 0.0026 after 5,000 permutation tests), which showed better performance than the other two models.

**Table 3:** Classification performance of machine methods.

	**AUC**	**Accuracy**	**Sensitivity**	**Sepecificity**	**Kappa**
SVM	0.78	0.71	0.82	0.60	0.47
RF	0.71	0.70	0.62	0.79	0.42
LR	0.77	0.71	0.84	0.58	0.43

SVM, support vector machine; RF, random forest; LR, logistic regression.

## Discussion

In this study, we extracted DC from the whole brain as a selection feature and combined it with a variety of machine learning methods (SVM, LR, RF) to train a classifier. We found that SVM had the best classification performance to distinguish OSA patients with cognitive impairment. Simultaneously, we also used LASSO to select the most discriminating brain regions used to distinguish MCI from nMCI, including the olfactory cortex, cingulate gyrus, globus pallidus, transverse temporal gyrus, cerebellum and other regions, providing more evidence to explain the heterogeneity and complexity of OSA patients with cognitive impairment.

### Machine learning

Due to the overall scarcity and financial burden of PSG, methods combining multidimensional clinical parameters and machine learning have been widely used to distinguish OSA, OSA severity, and OSA prognosis (2, 38, 39). However, studies using DC to distinguish OSA are relatively rare. Yujun Gao et al. (40) showed that compared with healthy people, changes in DC values of right superior frontal gyrus, hippocampus, superior temporal gyrus and caudate nucleus in epileptic patients can be distinguished with high precision between epileptic patients and healthy controls by combining SVM model, and the unique DC model can be used as an imaging marker for the diagnosis of epilepsy. Chang Xi et al. used whole-brain voxel level DC combined with machine learning to distinguish major depression and bipolar disorder. The DC reduction of default mode network and sensorimotor network can be used as an effective feature to distinguish depression, and the DC-based classification model has a high accuracy (91%) (41). These studies suggest that changes in DC can be used as neuroimaging markers to distinguish cognitive dysfunction.

LASSO is efficient for feature selection, avoiding data redundancy while preserving the most discriminating important features (42). Features were reduced according to LASSO, and the addition of reduction can improve model performance in partitioned OSA patients with and without MCI by avoiding overfitting and miscalibration. Altogether, among all the classifiers, the performance of SVM classifier is significantly better than other classifiers. Yu Zhou et al.(43) showed that the extraction of the white matter connection network in the hippocampus was used as an effective feature to classify the MCI group of AD patients and the healthy control group, SVM rbf classification efficiency (ACC = 89.4%, AUC = 0.954) was better than KNN (ACC = 86.9%, AUC = 0.920) and RF (ACC = 84.8%, AUC = 0.935). Based on DC features, this study uses three machine learning techniques to generate classification models, namely SVM, LR and RF. Since this study relied on a small data set, to obtain more sufficient data training, we adopted the keep-one method to test the model performance. Finally, SVM had the best classification performance (accuracy = 71%, AUC = 0.78), RF (accuracy = 70%, AUC = 0.71), LR (accuracy = 71%, AUC = 0.77). At the same time, linear kernel SVM can extract the weight of each feature and reflect the importance of each feature in the model.

### DC feature

The characteristics of screening between MCI and nMCI groups mainly involve the default network, basal ganglia area network, and cerebellar network. The DMN consists of discrete and bilaterally symmetric cortical regions, mainly involving the anteromedial frontal, temporal, and parietal cortex regions, and is characterized by high activity when the brain is not involved in tasks (44). Abnormal activity of the DMN is associated with cognitive function and symptoms of neuropsychiatric diseases (45, 46), and its functional changes have also been confirmed in relevant studies on OSA (47). Our previous study showed (48) that, based on the graph theory approach, DMN topological abnormalities in OSA patients were associated with cognitive dysfunction, especially memory delay and memory retrieval. Prilipko et al. showed that functional inactivation of the DMN region in OSA patients was significantly related to behavioral performance and episodic memory compared with the healthy group (49). These studies suggest that abnormal functional changes in the DMN may be one of the most effective markers to distinguish OSA patients with concomitant MCI.

The globus pallidus is one of the components of the basal ganglia and is involved in the final output of direct and indirect pathways of the basal ganglia network, and its impairment can cause a variety of cognitive and motor problems (50). Previous studies have confirmed that globus pallidus neurons have higher energy requirements and are more susceptible to oxygen deprivation than other neurons in the basal ganglia region (51). Oxidative stress is also one of the important factors of neuron degeneration in the basal ganglia (52). A study on the structure of the basal ganglia region found morphological changes in the left globus pallidus and thalamus in children with OSA (53), which is helpful for understanding the autonomic activity and respiratory muscle activity abnormalities caused by OSA-related dysfunction of the globus pallidus. Our findings suggest that DC differences in the globus pallidus contribute to our understanding of the neuroimaging mechanisms by which OSA leads to cognitive dysfunction, which may help distinguish OSA from MCI.

The cerebellum and extra-cerebellar structures have extensive connections in motor and non-motor aspects. The cerebellum is not only involved in motor control, but also in cognitive and affective processing (54), which is based on the anatomical basis that there are multiple parallel circuits in the cerebellum and the cerebral cortex that are widely interconnected (55). Previous studies have shown that OSA can lead to significant changes in the structure and function of cerebellum (56), and lack of sleep is also a risk factor for damaging cerebellum function (57). Our previous studies have shown increased intrinsic connectivity in the right posterior cerebellar lobe in OSA patients prior to treatment, which may be a functional compensation for chronic intermittent hypoxia (6). It has been proposed that changes in the internal function of the cerebellum can be used as a model to predict motor and cognitive tasks (58). Therefore, we believe that neuroimaging changes in the cerebellar network may be one of the effective markers for identifying OSA with cognitive impairment.

## Limitation

The current study has some limitations. First, the sample size was relatively small and there was no external data set to verify to improve the generalization ability of the model. Secondly, most of our participants were male OSA patients with severe OSA, which may not be applicable to OSA patients with mild OSA or female OSA patients. Finally, we discuss only one fMRI functional feature, which will be combined with other fMRI features (functional connectivity, gray matter volume) and highly relevant clinical features (such as hyperlipidemia) to improve the classification efficiency of the model in the future.

## Conclusion

Our results demonstrate an effective machine learning approach that uses DC as a feature to effectively identify OSA patients with concomitant cognitive impairment. This study helps us better understand the neuroimaging mechanisms of OSA causing cognitive impairment.

## Data availability statement

The raw data supporting the conclusions of this article will be made available by the authors, without undue reservation.

## Ethics statement

The studies involving human participants were reviewed and approved by Medical Ethics Committee of the First Affiliated Hospital of Nanchang University [2020(94)]. The patients/participants provided their written informed consent to participate in this study.

## Author contributions

XL and YS wrote, reviewed, and revised the manuscript. DP guided and designed the MRI experiment. HL analyzed the resting-state fMRI data. XL and HL analyzed and discussed the ideas of the paper. PY analyzed machine learning. WD, KL, YZ, and WX collected resting-state fMRI data and applied for the ethics approval. All authors contributed to the article and approved the submitted version.

## Funding

This study was supported by the National Natural Science Foundation of China (Grant No. 81860307), the Natural Science Foundation Project of Jiangxi, China (Grant Nos. 20202BABL216036 and 20181ACB20023), and Education Department Project of Jiangxi provincial, China (Grant No. GJJ190133).

## Conflict of interest

The authors declare that the research was conducted in the absence of any commercial or financial relationships that could be construed as a potential conflict of interest.

## Publisher's note

All claims expressed in this article are solely those of the authors and do not necessarily represent those of their affiliated organizations, or those of the publisher, the editors and the reviewers. Any product that may be evaluated in this article, or claim that may be made by its manufacturer, is not guaranteed or endorsed by the publisher.
